# Risk Prediction Model for Synchronous Oligometastatic Non-Small Cell Lung Cancer: Thoracic Radiotherapy May Not Prolong Survival in High-Risk patients

**DOI:** 10.3389/fonc.2022.897329

**Published:** 2022-07-15

**Authors:** Chunliu Meng, Fang Wang, Jia Tian, Jia Wei, Xue Li, Kai Ren, Liming Xu, Lujun Zhao, Ping Wang

**Affiliations:** ^1^ Department of Radiation Oncology, Tianjin Medical University Cancer Institute and Hospital, National Clinical Research Center for Cancer, Tianjin Key Laboratory of Cancer Prevention and Therapy, Tianjin's Clinical Research Center for Cancer, Tianjin, China; ^2^ Department of Radiation Oncology, Affiliated Hospital of Hebei University, Baoding, China; ^3^ Department of Oncology, Shandong Provincial Third Hospital, Shandong University, Jinan, China

**Keywords:** synchronous oligometastasis, non–small cell lung cancer, thoracic radiotherapy, risk prediction model, survival

## Abstract

**Background and Purpose:**

On the basis of the promising clinical study results, thoracic radiotherapy (TRT)^1^ has become an integral part of treatment of synchronous oligometastatic non–small cell lung cancer (SOM-NSCLC). However, some of them experienced rapid disease progression after TRT and showed no significant survival benefit. How to screen out such patients is a more concerned problem at present. In this study, we developed a risk-prediction model by screening hematological and clinical data of patients with SOM-NSCLC and identified patients who would not benefit from TRT.

**Materials and Methods:**

We investigated patients with SOM-NSCLC between 2011 and 2019. A formula named Risk-Total was constructed using factors screened by LASSO-Cox regression analysis. Stabilized inverse probability treatment weight analysis was used to match the clinical characteristics between TRT and non-TRT groups. The primary endpoint was overall survival (OS).

**Results:**

We finally included 283 patients divided into two groups: 188 cases for the training cohort and 95 for the validation cohort. Ten prognostic factors included in the Risk-Total formula were age, N stage, T stage, adrenal metastasis, liver metastasis, sensitive mutation status, local treatment status to metastatic sites, systemic inflammatory index, CEA, and Cyfra211. Patients were divided into low- and high-risk groups based on risk scores, and TRT was found to have improved the OS of low-risk patients (46.4 vs. 31.7 months, *P =* 0.083; 34.1 vs. 25.9 months, *P =* 0.078) but not that of high-risk patients (14.9 vs. 11.7 months, *P =* 0.663; 19.4 vs. 18.6 months, *P =* 0.811) in the training and validation sets, respectively.

**Conclusion:**

We developed a prediction model to help identify patients with SOM-NSCLC who would not benefit from TRT, and TRT could not improve the survival of high-risk patients.

## Introduction

Non–small cell lung cancer (NSCLC)^2^ is a common malignant tumor that accounts for 70%–80% of all lung cancer cases worldwide. NSCLC is associated with high morbidity and mortality rates (1). More than half of patients with NSCLC have stage IV disease at the time of diagnosis, and up to one-third of these patients have synchronous oligometastatic (SOM) disease (2, 3).

SOM disease has been described as a distinct disease entity characterized by reduced metastatic potential with a limited number of metastatic sites (4), which renders it amenable to local treatment (LT). There is no consensus on what specific criteria define SOM-NSCLC. Of note, inclusion criteria for previously cited studies were very different. Recently, the European Organization for Research and Treatment of cancer (EORTC) and the European Society of Radiotherapy & Oncology-American Society for Therapeutic Radiology and Oncology (ESTRO-ASTRO) conferences had attempted to standardize the definition of oligometastatic disease (2, 5). The documents showed that the definition of oligometastatic disease should base on safety of radical treatment rather than the number of metastases, and it would be better the number of metastatic lesions ≤ 5 and the number of metastatic sites ≤ 3, with or without primary sites, and mediastinal metastatic lymph nodes were included. Several clinical trials and multiple retrospectives series have reported favorable outcomes of thoracic radiotherapy (TRT) in highly selected patients with SOM-NSCLC (6–14). However, some of them experienced rapid disease progression after TRT and showed no significant survival benefit. And, to date, no effective predictive model has been developed to help identify patients with SOM-NSCLC who would not benefit from TRT. In this study, we sought to establish a risk prediction model to predict the mortality risk of these patients using baseline hematologic and clinical data and to identify patients who would not benefit from TRT.

## Materials and methods

### Patient Selection

We retrospectively reviewed the medical records of consecutive patients who received a diagnosis of advanced NSCLC at our hospital between January 2011 and December 2019. Clinical staging of the disease at the time of presentation was again determined with reference to the eighth edition of tumor node metastasis classification (15). The inclusion criteria for this study were as follows: (1) confirmed diagnosis of NSCLC based on pathological or cytological specimens, or both; (2) patients were allowed to have up to five lesions of metastatic disease (do not include primary site and enlarged lymph nodes in the mediastinum and supraclavicular) with no more than three sites (2, 5); and (3) availability of gene mutation status information. To determine metastasis status, patients needed to undergo comprehensive imaging tests, including head contrast-enhanced MRI, neck ultrasound, chest–abdomen contrast-enhanced CT plus ECT, or PET-CT. If there was ambiguous metastatic lesion in the liver, then contrast-enhanced abdominal MRI was also necessary. Meanwhile, patients were excluded if they had second primary tumor, pleural or pericardial effusion, meningeal or peritoneal metastases, a metastatic site with ambiguous diagnosis, or incomplete medical records.

### Definition of Special Concept

In this study, positively sensitive mutations (SM^+^) included the following: *EGFR* (epidermal growth factor receptor) exon 19 deletion, *EGFR* exon 21 Leu858Arg mutation, *ROS* proto-oncogene 1, receptor tyrosine kinase (*ROS1*) fusion mutation, and *ALK* (anaplastic lymphoma kinase) mutation. *EGFR* uncommon mutations, such as exon 18 mutations, exon 20 insertion mutations, and so on, and other non-targeted therapeutic mutations or without any mutation, were defined as sensitive mutation negative (SM^−^).

### Hematological Markers

Laboratory examinations including routine blood tests, hepatic and renal function tests, and tumor markers of patients were collected before initial treatment. The calculation formulas of neutrophil-to-lymphocyte ratio (NLR), platelet-to-lymphocyte ratio (PLR), and systemic inflammatory index (SII) were as follows: NLR = neutrophil number (10^9^/L)/lymphocyte count (10^9^/L); PLR = number of platelets (10^9^/L)/number of lymphocytes (10^9^/L); SII = number of platelets (10^9^/L) × number of neutrophils (10^9^/L)/number of lymphocytes (10^9^/L). The optimal cutoff levels for albumin, leukocyte, PLR, NLR, SII, tissue polypeptide–specific antigen (TPSA), squamous cell carcinoma antigen (SCC), Ca19-9, carcinoembryonic antigen (CEA), and Cyfra211 were obtained according to overall survival (OS).

### Thoracic Radiotherapy

In this study, 150 patients received TRT, and TRT could be carried out before, concomitant or after the systemic treatment. The specific radiotherapy target was determined by patient’s attending physician. Generally, gross tumor volume (GTV) included primary lesions with or without mediastinal metastatic lymph nodes, and planning GTV (PGTV) extends 5 mm across the GTV margin. Radiation therapy technology could apply conventional fractionated radiotherapy, hypo-fractionated radiotherapy, and stereotactic body radiotherapy, and the radiation doses were 1.8–2.1 Gy/50–66 Gy, 3 Gy/36–45 Gy, and 9–17 Gy/50–60 Gy, respectively.

### First-line Systemic Treatment Strategy

All patients with *EGFR* non-SMs, untargeted therapy mutations or without mutation, underwent first-line chemotherapy after confirmation of the initial NSCLC diagnosis. The treatment included platinum-based doublet chemotherapy such as pemetrexed, paclitaxel, docetaxel, or gemcitabine combined with cisplatin, carboplatin, or nedaplatin. Each chemotherapy session was separated by an interval of 3 to 4 weeks.

Patients with *EGFR*-SMs (exon 19 deletion, exon 21 Leu858Arg mutations) were administered first-line treatment with *EGFR* tyrosine kinase inhibitors (TKIs), such as gefitinib, erlotinib, and icotinib, or with chemotherapy mentioned above and then TKIs after disease progression. Patients with *ALK* and *ROS1* mutation were administered first-line treatment with crizotinib or with chemotherapy as aforesaid and then TKIs after disease progression.

### Data Analysis and Statistical Considerations

The primary endpoint was OS defined as the time from the date of diagnosis until death or the most recent follow-up. The follow-up schedule began from the time of treatment to the final follow-up on December 17, 2021. The data on the date of death or at the final follow-up visit were acquired from hospital records or through direct correspondence with the family of the patient. R 4.1.1 and SPSS 24.0 software were used for statistical analyses. The Chi-squared test (or the Fisher’s exact test as applicable) was used to compare the clinical characteristics between groups. OS was estimated using the Kaplan–Meier method, and between-group differences in OS were assessed using the log-rank test. The optimal cutoff values of hematological markers were determined using the package “survminer” based on OS. Using the “glmnet” and “survival” packages and a backward–forward stepwise method, LASSO-Cox regression analysis was performed to select the optimal prognostic factors. The “predict” function of package “survival” was used to calculate the risk score of each patient. Time-dependent receiver operator characteristic (ROC) analyses were conducted using the “timeROC” package. Package “IPWsurvival” was used for stabilized inverse probability treatment weight (IPTW) analyses.

## Results

### Patient Characteristics

This study had been approved by the Ethics Committee of Tianjin Medical University Cancer Hospital (ab2022138). A total of 2,194 patients were diagnosed with advanced NSCLC at our hospital during the study reference period. Of these, 1,624, 23, 54, 76, and 134 patients were excluded due to extensive metastatic lesions, second primary tumors, pleural effusion, lack of gene sequencing results, and incomplete medical records, respectively.

Finally, 283 patients with SOM-NSCLC fulfilled the inclusion criteria for this study. The median OS was 23.4 months, and the 1-, 3-, and 5-year OS rates were 73.3%, 30.1%, and 11.5%, respectively. The entire cohort was randomly divided into two groups by a ratio of 2:1, 188 cases in the training set and 95 cases in the validation set, respectively. The median OS were 22.7 and 24.4 months, respectively; and 1-, 3-, and 5-year OS rates were 72.1%, 31.4%, and 12.7% and 75.6%, 27.0%, and 9.1%, respectively; and there was no difference in survival between sets (*P =* 0.655). The patient characteristics were summarized in **Table 1**.

**Table 1:** Clinical characteristics of patients.

Characteristics	Training set (N=188)		Validation set (N=95)	*P* value
	No. of patients (%)		No. of patients (%)	
Age				0.266
<65	116 (61.7)		65 (68.4)	
≥65	72 (38.3)		30 (31.6)	
Mean ± SD	61.2 ± 9.28		60.0 ± 8.11	0.282
Sex				0.082
Male	134 (71.3)		58 (61.1)	
Female	54 (28.7)		37 (38.9)	
KPS				0.773
<80	14 (7.4)		8 (8.4)	
≥80	174 (92.6)		87 (91.6)	
Smoking				0.017*
No	71 (37.8)		50 (52.6)	
Yes	117 (62.2)		45 (47.4)	
Histopathology				0.328
Adenocarcinoma	130 (69.1)		71 (74.7)	
Non-adenocarcinoma	58 (30.9)		24 (25.3)	
N stage				0.253
N0	47 (25.0)		18 (18.9)	
N1-3	141 (75.0)		77 (81.1)	
T stage				0.282
T1-2	125 (66.5)		57 (60.0)	
T3-4	63 (33.5)		38 (40.0)	
SM				0.153
Yes	61 (32.4)		39 (41.1)	
No	127 (67.6)		56 (8.9)	
LT status to metastatic sites before PD				0.764
All	60 (31.9)		32 (33.7)	
Partly or no	128 (68.1)		63 (66.3)	
Brain metastasis	38 (20.2)		13 (13.7)	0.177
Bone metastasis	82 (43.6)		48 (50.5)	0.271
Adrenal metastasis	22 (11.7)		9 (9.5)	0.571
Liver metastasis	5 (2.7)		1 (1.1)	0.653
TRT				0.968
CFR	49 (26.1)		23 (24.2)	
HFR	15 (8.0)		7 (7.4)	
SBRT	37(19.7)		19 (20.0)	
Albumin (g/L)	42.1 ± 4.19		41.1 ± 3.88	0.060
Leukocyte (10^9^/L)	7.6 ± 2.46		7.3 ± 1.99	0.366
PLR	171.8 ± 92.61		165.3 ± 72.76	0.551
NLR	3.1 ± 2.05		3.2 ± 3.18	0.730
SII	888.0 ± 675.57		836.1 ± 572.67	0.522
TPSA (U/L)	114.2 ± 209.5		130.4 ± 222.36	0.548
SCC (µg/L)	2.5 ± 6.92		2.5 ± 6.95	0.926
Ca19-9 (U/mL)	40.1 ± 82.74		52.9 ± 153.82	0.366
CEA (µg/L)	39.6 ± 106.26		73.9 ± 183.17	0.094
Cyfra211 (µg/L)	6.6 ± 9.13		11.2 ± 23.00	0.064

*P<0.05.

KPS, Karnofsky performance status; SM, sensitive mutation; LT, local treatment; PD, progress disease; TRT, thoracic radiotherapy; CFR, conventional fractionated radiotherapy; HFR, hypo-fractionated radiotherapy; SBRT, stereotactic body radiotherapy; PLR, platelet to lymphocyte ratio; NLR, neutrophils to lymphocyte ratio; SII, systemic inflammatory index; TPSA, tissue polypeptide specific antigen; SCC, squamous cell carcinoma antigen; CEA, carcinoembryonic antigen.

### Construction of Risk-Total Formula in the Training Set

In the training set, hematological markers, including albumin, leukocyte, PLR, NLR, SII, TPSA, SCC, Ca199, CEA, and Cyfra211, were divided into low and high groups according to the respective optimal cutoff levels (**Table 2**).

**Table 2:** Cutoff level and univariate Cox analyses of hematological markers in the training set.

Characteristics	Cutoff	Categories	*P* value
Albumin	45.40	High (≥ 45.40) vs. Low (< 45.40)	0.014*
Leukocyte	7.82	High (≥ 7.82) vs. Low (< 7.82)	0.009*
PLR	112.24	High (≥112.24) vs. Low (<112.24)	0.096
NLR	1.63	High (≥ 1.63) vs. Low (< 1.63)	0.007*
SII	366.36	High (≥ 366.36) vs. Low (< 366.36)	0.001*
TPSA	95.56	High (≥ 95.56) vs. Low (< 95.56)	0.001*
SCC	1.60	High (≥ 1.60) vs. Low (< 1.60)	< 0.001*
Ca19-9	7.45	High (≥ 7.45) vs. Low (< 7.45)	0.197
CEA	2.00	High (≥ 2.00) vs. Low (< 2.00)	0.009*
Cyfra211	3.71	High (≥ 3.71) vs. Low (< 3.71)	< 0.001*

*P<0.05.

PLR, platelet to lymphocyte ratio; NLR, neutrophils to lymphocyte ratio; SII, systemic inflammatory index; TPSA, tissue polypeptide specific antigen; SCC, squamous cell carcinoma antigen; CEA, carcinoembryonic antigen.

To assess the mortality risk of each patient in the training set, we established a prognostic scoring system named Risk-Total using LASSO-Cox regression model (**Figure A.1**). Hematological markers mentioned above and other clinical variables, such as age, sex, Karnofsky performance status (KPS), smoking, histopathology, T stage, N stage, brain metastasis, bone metastasis, adrenal metastasis, liver metastasis, SM status, and LT status to metastatic site status before progression disease (PD), were included in the analysis. In this model, low albumin, high leukocyte, high PLR, high NLR, high SII, high TPSA, high SCC, high Ca199, high CEA, high Cyfra211, age ≥ 65, male, KPS < 80, smoking, N1–3, T3–4, non-adenocarcinoma, presence of brain metastasis, bone metastasis, adrenal metastasis, liver metastasis, SM^−^, and metastatic sites receiving partial or no LT before PD were assigned in level 2, and the corresponding alternatives were assigned in level 1.

**Figure 1:**
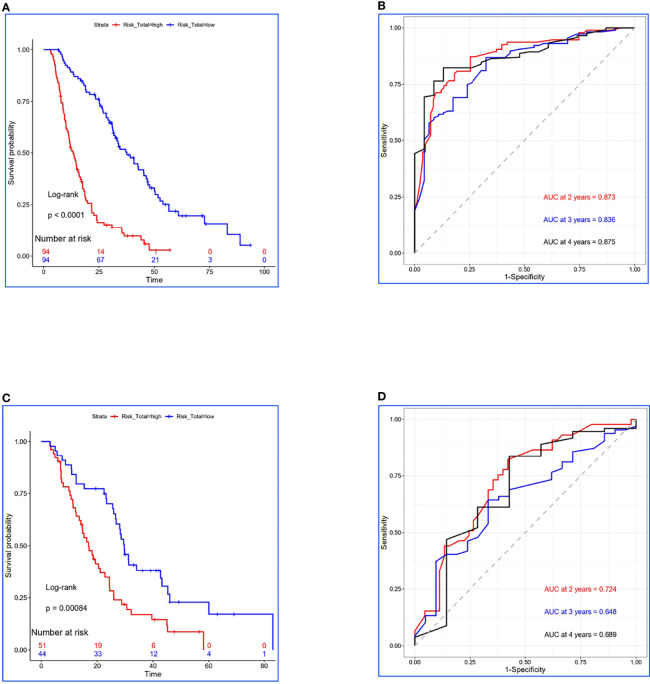
Construction and validation for Risk-Total. **(A, C)** Kaplan–Meier survival analyses of Risk-Total in the training set and the validation set. **(B, D)** Risk-Total performance in time-dependent receiver operating characteristic (ROC) curves in the training set and the validation set.

Finally, 10 variables were included in the optimal model (AIC = 1,251.94, *P* < 2.2 × 10^−16^) as follows: Risk-Total = 1 × HR-value (age) × HR-value (N stage) × HR-value (T stage) × HR-value (adrenal metastasis) × HR-value (liver metastasis) × HR-value (SM status) × HR-value (LT status to metastatic sites before PD) × HR-value (SII) × HR-value (CEA) × HR-value (Cyfra211) (**Table 3**). According to the median Risk-Total value (10.0658), patients were divided into low-risk and high-risk groups, and the median survival time (MST) were 37.6 and 13.4 months, respectively (*P* < 0.001; **Figure 1A**). Meanwhile, the prognostic accuracy of Risk-Total was evaluated by time-dependent ROC analyses, with 2-, 3-, and 4-year AUC values of 0.873, 0.836, and 0.875, respectively, which confirmed the excellent prognostic power of it (**Figure 1B**). The patient characteristics between low- and high-risk groups were displayed in **Table 4**.

**Table 3:** Factors included in the Risk-Total formula.

Characteristics	Level	Coefficient	HR-value	*P* value
Age	1 = <65	0.3372	1	0.05597
	2 = ≥65		1.4010	
N stage	1 = N0	0.3463	1	0.08476
	2 = N1-3		1.4138	
T stage	1 = T1-2	0.4127	1	0.02272*
	2 = T3-4		1.5109	
Adrenal metastasis	1 = no	0.4580	1	0.06697
	2 = yes		1.5810	
Liver metastasis	1 = no	1.0923	1	0.02658*
	2 = yes		2.9811	
SM status	1 = SM^+^	0.8548	1	9.09×e-06*
	2 = SM^−^		2.3510	
LT status to metastatic sites before PD	1 = All	0.5407	1	0.00505*
	2 = Partly or no		1.7172	
SII	1 = low	0.9098	1	0.00348*
	2 = high		2.4838	
CEA	1 = low	-0.6275	1	0.01300*
	2 = high		0.5339	
Cyfra211	1 = low	0.8142	1	1.19×e-05*
	2 = high		2.2574	

*P<0.05.

SM, sensitive mutation; PD, progress disease; SII, systemic inflammatory index; CEA, carcinoembryonic antigen.

Risk-Total = 1*HR-value (age) *HR-value (N stage) *HR-value (T stage) *HR-value (adrenal metastasis) *HR-value (liver metastasis) *HR-value (SM status) *HR-value (LT to metastatic sites status before PD) *HR-value (SII) *HR-value (CEA) *HR-value (Cyfra211).

**Table 4:** Clinical characteristics of low- and high-risk patients in the training set.

Characteristics	Low risk (N=94)	High risk (N=94)	*P* value
	No. of patients (%)	No. of patients (%)	
Age			0.134
<65	63 (67.0)	53 (56.4)	
≥65	31 (33.0)	41 (43.6)	
Mean ± SD	59.6 ± 9.76	62.8 ± 8.53	0.017*
Sex			0.010*
Male	59 (62.8)	75 (79.8)	
Female	35 (37.2)	19 (20.2)	
KPS			0.578
<80	8 (8.5)	6 (6.4)	
≥80	86 (91.5)	88 (93.6)	
Smoking			0.001*
No	47 (50.0)	24 (25.5)	
Yes	47 (50.0)	70 (74.5)	
Histopathology			0.001*
Adenocarcinoma	76 (80.9)	54 (57.4)	
Non-adenocarcinoma	18 (19.1)	40 (42.6)	
N stage			0.029*
N0	30 (31.9)	17 (18.1)	
N1-3	64 (68.9)	77 (81.9)	
T stage			<0.001*
T1-2	77 (81.9)	48 (51.1)	
T3-4	17 (19.1)	46 (48.9)	
SM			<0.001*
Yes	50 (53.2)	11 (41.1)	
No	44 (46.8)	83 (8.9)	
LT status to metastatic sites before PD			0.001*
All	41 (43.6)	19 (20.2)	
Partly or no	53 (56.4)	75 (79.8)	
Brain metastasis	21 (22.3)	17 (18.1)	0.468
Bone metastasis	48 (51.1)	34 (36.2)	0.039*
Adrenal metastasis	4 (4.3)	18 (19.1)	0.001*
Liver metastasis	1 (1.1)	4 (4.3)	0.365
TRT			0.283
CFR	22 (23.4)	27 (28.7)	
HFR	8 (8.5)	7 (7.4)	
SBRT	23 (24.5)	14 (14.9)	
Albumin (g/L)	42.5 ± 4.64	41.7 ± 3.67	0.165
Leukocyte (10^9^/L)	7.4 ± 2.74	7.8 ± 2.13	0.304
PLR	163.1 ± 63.81	180.5 ± 114.12	0.200
NLR	3.2 ± 2.45	3.0 ± 1.57	0.507
SII	838.4 ± 570.29	937.6 ± 766.49	0.316
TPSA (U/L)	91.7 ± 144.8	136.8 ± 257.35	0.140
SCC (µg/L)	1.8 ± 7.19	3.2 ± 6.61	0.165
Ca19-9 (U/mL)	30.8 ± 61.31	49.5 ± 99.15	0.122
CEA (µg/L)	39.6 ± 106.26	73.9 ± 183.17	0.130
Cyfra211 (µg/L)	5.1 ± 8.39	8.0 ± 9.64	0.027*

*P<0.05.

KPS, Karnofsky performance status; SM, sensitive mutation; LT, local treatment; PD, progress disease; TRT, thoracic radiotherapy; CFR, conventional fractionated radiotherapy; HFR, hypo-fractionated radiotherapy; SBRT, stereotactic body radiotherapy; PLR, platelet to lymphocyte ratio; NLR, neutrophils to lymphocyte ratio; SII, systemic inflammatory index; TPSA, tissue polypeptide specific antigen; SCC, squamous cell carcinoma antigen; CEA, carcinoembryonic antigen.

### Validation of Risk-Total Formula in the Validation Set

In the validation set, patients’ hematological markers were grouped on the basis of cutoff value, as shown in **Table 2**, and the risk score were calculated on the basis of Risk-Total formula, as shown in **Table 3**. Then, according to the median value (10.0658) mentioned above, patients were divided into low-risk and high-risk groups, and the MST were 29.7 and 16.9 months, respectively (*P =* 0.00084; **Figure 1C**). Similarly, the prognostic accuracy of Risk-Total was also evaluated by time-dependent ROC analyses, with 2-, 3-, and 4-year AUC values of 0.724, 0.648, and 0.689, respectively (**Figure 1D**). These results confirmed the super prognostic power of Risk-Total in another heterogeneous population. The patient characteristics between low- and high-risk groups are shown in **Table 5**.

**Table 5:** Clinical characteristics of low- and high-risk patients in the validation set.

Characteristics	Low risk (N=44)	High risk (N=51)	*P* value
	No. of patients (%)	No. of patients (%)	
Age			0.085
<65	34 (77.3)	31 (60.8)	
≥65	10 (22.7)	20 (39.2)	
Mean ± SD	58.5 ± 7.47	61.3 ± 8.47	0.092
Sex			0.227
Male	24 (54.5)	34 (66.7)	
Female	20 (45.5)	17 (33.3)	
KPS			1.000
<80	4 (9.1)	4 (7.8)	
≥80	40 (90.9)	47 (92.2)	
Smoking			0.046*
No	28 (63.6)	22 (43.1)	
Yes	16 (36.4)	29 (56.9)	
Histopathology			0.051
Adenocarcinoma	37 (84.1)	34 (66.7)	
Non-adenocarcinoma	7 (15.9)	17 (33.3)	
N stage			0.054
N0	12 (27.3)	6 (11.8)	
N1-3	32 (72.7)	45 (88.2)	
T stage			<0.001*
T1-2	36 (81.8)	21 (41.2)	
T3-4	8 (18.2)	30 (58.8)	
SM			<0.001*
Yes	31 (70.5)	8 (15.7)	
No	13 (29.5)	43 (84.3)	
LT status to metastatic sites before PD			0.007*
All	21 (47.7)	11 (21.6)	
Partly or no	23 (52.3)	40 (78.4)	
Brain metastasis	7 (15.9)	6 (11.8)	0.558
Bone metastasis	29 (65.9)	19 (37.3)	0.005*
Adrenal metastasis	1 (2.3)	8 (15.7)	0.061
Liver metastasis	1 (2.3)	0 (0.0)	0.941
TRT			0.227
CFR	11 (25.0)	12 (23.5)	
HFR	4 (9.1)	3 (5.9)	
SBRT	11 (31.8)	5 (9.8)	
Albumin (g/L)	41.4 ± 3.75	40.8 ± 4.00	0.454
Leukocyte (10^9^/L)	7.1 ± 2.08	7.6 ± 1.91	0.234
PLR	159.1 ± 70.64	170.7 ± 74.81	0.440
NLR	2.9 ± 2.18	3.5 ± 3.85	0.346
SII	790.2 ± 665.89	875.6 ± 481.41	0.472
TPSA (U/L)	81.9 ± 98.81	172.3 ± 284.07	0.037*
SCC (µg/L)	1.3 ± 1.79	3.5 ± 9.27	0.109
Ca19-9 (U/mL)	29.0 ± 61.30	52.9 ± 153.82	0.139
CEA (µg/L)	39.6 ± 106.26	73.4 ± 200.80	0.108
Cyfra211 (µg/L)	5.8 ± 9.64	15.8 ± 29.46	0.025*

*P<0.05.

KPS, Karnofsky performance status; SM, sensitive mutation; LT, local treatment; PD, progress disease; TRT, thoracic radiotherapy; CFR, conventional fractionated radiotherapy; HFR, hypo-fractionated radiotherapy; SBRT, stereotactic body radiotherapy; PLR, platelet to lymphocyte ratio; NLR, neutrophils to lymphocyte ratio; SII, systemic inflammatory index; TPSA, tissue polypeptide specific antigen; SCC, squamous cell carcinoma antigen; CEA, carcinoembryonic antigen.

### Prognostic Value of TRT for Low- and High- risk Patients

**Figure 2:**
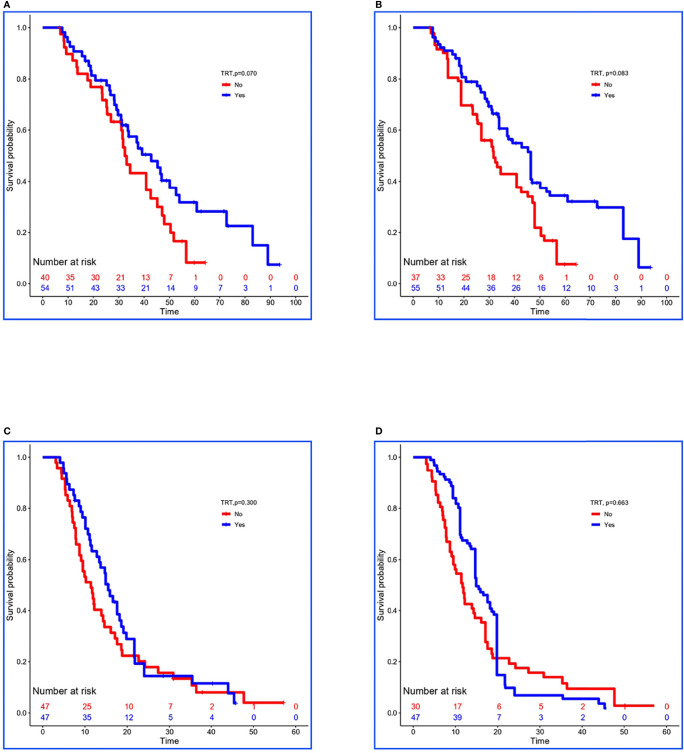
Kaplan–Meier survival analyses for patients between groups. **(A, B)** Survival curves for low-risk patients between non-TRT and TRT groups when clinical characteristics were unmatched and matched using stabilized IPTW analysis in the training set. **(C, D)** Survival curves for high-risk patients between non-TRT and TRT groups when clinical characteristics were unmatched and matched using stabilized IPTW analysis in the training set.

In the training set, 54 of 94 patients with low-risk received TRT, and survival analysis showed improvement in OS (42.8 vs. 32.4 months, *P =* 0.070; **Figure 2A**). However, the inter-group clinical characteristics were very unbalanced, especially with respect to age, gender, LT status to metastatic sites, and PLR (**Table 6A**). Therefore, we applied the stabilized IPTW analysis to calculate the weights of clinical variables and match them. After matching, TRT was still found to improve the OS (46.4 vs. 31.7 months, *P =* 0.083; **Figure 2B**). Whereas, 47 of 94 patients with high-risk received TRT, but the OS was not prolonged (15.5 vs. 11.4 months, *P =* 0.300; **Figure 2C**). When the clinical variables were calculated weights and matched (**Table 6B**), the survival time was not improved all the same (14.9 vs. 11.7 months, *P =* 0.663; **Figure 2D**).

**Table 6:** Comparison of clinical characteristics of patients in no-TRT and TRT subgroups.

A								
		Unmatched				Stabilized IPTW		
	Level	no-TRT (%)	TRT (%)	*P*		no-TRT (%)	TRT (%)	*P*
Number		40	54			36.8	55	
Age	<65	33 (82.5)	32 (59.3)	0.029		25.6 (69.6)	38.8 (70.5)	0.937
	>=65	7 (17.5)	22 (40.7)			11.2 (30.4)	16.2 (29.5)	
Gender	female	20 (50.0)	14 (25.9)	0.029		15.1 (41.1)	22.2 (40.3)	0.952
	male	20 (50.0)	40 (74.1)			21.7 (58.9)	32.8 (59.7)	
KPS	>=80	37 (92.5)	49 (90.7)	1.000		34.8 (94.5)	51.0 (92.8)	0.723
	<80	3 (7.5)	5 (9.3)			2.0 (5.5)	4.0 (7.2)	
Smoking	no	21 (52.5)	25 (46.3)	0.699		18.0 (48.8)	30.6 (55.7)	0.594
	yes	19 (47.5)	29 (53.7)			18.8 (51.2)	24.4 (44.3)	
Histopathology	adenocarcinoma	36 (90.0)	44 (81.5)	0.393		29.1 (79.0)	46.2 (84.0)	0.651
	non-adenocarcinoma	4 (10.0)	10 (18.5)			7.7 (21.0)	8.8 (16.0)	
N stage	N0	10 (25.0)	23 (42.6)	0.122		11.8 (32.1)	18.3 (33.3)	0.921
	N1-3	30 (75.0)	31 (57.4)			25.0 (67.9)	36.6 (66.7)	
T stage	T1-2	34 (85.0)	47 (87.0)	1.000		32.9 (89.4)	48.8 (88.7)	0.910
	T3-4	6 (15.0)	7 (13.0)			3.9 (10.6)	6.2 (11.3)	
Brain metastasis	no	31 (77.5)	39 (72.2)	0.733		29.4 (79.8)	43.0 (78.2)	0.869
	yes	9 (22.5)	15 (27.8)			7.4 (20.2)	12.0 (21.8)	
Bone metastasis	no	18 (45.0)	30 (55.6)	0.422		17.4 (47.3)	26.1 (47.5)	0.993
	yes	22 (55.0)	24 (44.4)			19.4 (52.7)	28.9 (52.5)	
Adrenal metastasis	no	37 (92.5)	52 (96.3)	0.729		35.1 (95.4)	52.8 (96.1)	0.882
	yes	3 (7.5)	2 (3.7)			1.7 (4.6)	2.2 (3.9)	
SM status	SM+	25 (62.5)	28 (51.9)	0.413		20.4 (55.6)	31.7 (57.7)	0.869
	SM-	15 (37.5)	26 (48.1)			16.4 (44.4)	23.3 (42.3)	
LT to metastatic sites before PD	all	13 (32.5)	31 (57.4)	0.029		13.5 (36.6)	23.3 (42.4)	0.641
	partly or no	27 (67.5)	23 (42.6)			23.3 (63.4)	31.7 (57.6)	
Albumin	high	8 (20.0)	14 (25.9)	0.671		6.5 (17.6)	10.9 (19.8)	0.790
	low	32 (80.0)	40 (74.1)			30.3 (82.4)	44.1 (80.2)	
Leukocyte	low	27 (67.5)	40 (74.1)	0.641		25.8 (70.0)	41.6 (75.7)	0.603
	high	13 (32.5)	14 (25.9)			11.1 (30.0)	13.3 (24.3)	
PLR	low	8 (20.0)	24 (44.4)	0.024		12.6 (34.2)	17.3 (31.4)	0.827
	high	32 (80.0)	30 (55.6)			24.2 (65.8)	37.7 (68.6)	
NLR	low	5 (12.5)	12 (22.2)	0.347		8.1 (22.1)	9.5 (17.2)	0.656
	high	35 (87.5)	42 (77.8)			28.7 (77.9)	45.5 (82.8)	
SII	low	6 (15.0)	16 (29.6)	0.159		9.5 (25.9)	13.4 (24.3)	0.901
	high	34 (85.0)	38 (70.4)			27.3 (74.1)	41.6 (75.7)	
TPSA	low	31 (77.5)	46 (85.2)	0.493		31.4 (85.4)	47.1 (85.7)	0.973
	high	9 (22.5)	8 (14.8)			5.4 (14.6)	7.9 (14.3)	
SCC	low	38 (95.0)	47 (87.0)	0.346		33.6 (91.4)	49.3 (89.8)	0.834
	high	2 (5.0)	7 (13.0)			3.2 (8.6)	5.6 (10.2)	
Ca199	low	11 (27.5)	13 (24.1)	0.891		7.4 (20.1)	10.1 (18.5)	0.846
	high	29 (72.5)	41 (75.9)			29.4 (79.9)	44.8 (81.5)	
CEA	low	0 (0.0)	4 (7.4)	0.214		0.0 (0.0)	2.3 (4.2)	0.135
	high	40 (100.0)	50 (92.6)			36.8 (100.0)	52.7 (95.8)	
Cyfra211	low	25 (62.5)	37 (68.5)	0.697		25.4 (69.1)	40.2 (73.1)	0.714
	high	15 (37.5)	17 (31.5)			11.4 (30.9)	14.8 (26.9)

**Table:** 

B								
		Unmatched				Stabilized IPTW		
	Level	no-TRT (%)	TRT (%)	*P*		no-TRT (%)	TRT (%)	*P*
Number		47	47			30.4	47.2	
Age	<65	28 (59.6)	23 (48.9)	0.408		16.9 (55.5)	27.2 (57.6)	0.893
	>=65	19 (40.4)	24 (51.1)			13.5 (44.5)	20.0 (42.4)	
Gender	female	14 (29.8)	6 (12.8)	0.078		7.4 (24.3)	3.4 (7.3)	0.018
	male	33 (70.2)	41 (87.2)			23.0 (75.7)	43.7 (92.7)	
KPS	>=80	44 (93.6)	44 (93.6)	1.000		28.2 (92.7)	43.9 (93.1)	0.949
	<80	3 (6.4)	3 (6.4)			2.2 (7.3)	3.2 (6.9)	
Smoking	no	15 (31.9)	10 (21.3)	0.350		8.5 (27.8)	6.1 (13.0)	0.092
	yes	32 (68.1)	37 (78.7)			21.9 (72.2)	41.1 (87.0)	
Histopathology	adenocarcinoma	30 (63.8)	20 (42.6)	0.063		19.7 (64.9)	27.9 (59.1)	0.697
	non-adenocarcinoma	17 (36.2)	27 (57.4)			10.7 (35.1)	19.3 (40.9)	
N stage	N0	3 (6.4)	11 (23.4)	0.043		2.0 (6.4)	6.0 (12.6)	0.317
	N1-3	44 (93.6)	36 (76.6)			28.5 (93.6)	41.2 (87.4)	
T stage	T1-2	22 (46.8)	22 (46.8)	1.000		13.2 (43.5)	22.3 (47.2)	0.820
	T3-4	25 (53.2)	25 (53.2)			17.2 (56.5)	24.9 (52.8)	
Brain metastasis	no	40 (85.1)	40 (85.1)	1.000		26.3 (86.5)	41.9 (88.9)	0.757
	yes	7 (14.9)	7 (14.9)			4.1 (13.5)	5.3 (11.1)	
Bone metastasis	no	29 (61.7)	29 (61.7)	1.000		16.9 (55.7)	18.1 (38.3)	0.236
	yes	18 (38.3)	18 (38.3)			13.5 (44.3)	29.1 (61.7)	
Adrenal metastasis	no	40 (85.1)	37 (78.7)	0.592		26.0 (85.4)	40.2 (85.2)	0.987
	yes	7 (14.9)	10 (21.3)			4.5 (14.6)	7.0 (14.8)	
Liver metastasis	no	44 (93.6)	45 (95.7)	1.000		28.8 (94.5)	46.0 (97.5)	0.392
	yes	3 (6.4)	2 (4.3)			1.7 (5.5)	1.2 (2.5)	
SM status	SM+	7 (14.9)	1 (2.1)	0.065		3.5 (11.5)	0.5 (1.1)	0.007
	SM-	40 (85.1)	46 (97.9)			26.9 (88.5)	46.7 (98.9)	
LT to metastatic sites before PD	all	1 (2.1)	15 (31.9)	0.001		0.6 (1.9)	7.6 (16.0)	0.015
	partly or no	46 (97.9)	32 (68.1)			29.8 (98.1)	39.6 (84.0)	
Albumin	high	4 (8.5)	4 (8.5)	1.000		2.5 (8.4)	2.5 (5.2)	0.533
	low	43 (91.5)	43 (91.5)			27.9 (91.6)	44.7 (94.8)	
Leukocyte	low	22 (46.8)	24 (51.1)	0.837		14.2 (46.8)	22.6 (47.9)	0.946
	high	25 (53.2)	23 (48.9)			16.2 (53.2)	24.6 (52.1)	
PLR	low	6 (12.8)	8 (17.0)	0.772		3.4 (11.2)	4.5 (9.5)	0.774
	high	41 (87.2)	39 (83.0)			27.0 (88.8)	42.7 (90.5)	
NLR	low	1 (2.1)	3 (6.4)	0.609		0.8 (2.6)	1.8 (3.8)	0.744
	high	46 (97.9)	44 (93.6)			29.6 (97.4)	45.4 (96.2)	
SII	low	0 (0.0)	3 (6.4)	0.241		0.0 (0.0)	1.5 (3.2)	0.260
	high	47 (100.0)	44 (93.6)			30.4 (100.0)	45.7 (96.8)	
TPSA	low	26 (55.3)	28 (59.6)	0.835		17.4 (57.2)	27.3 (57.8)	0.971
	high	21 (44.7)	19 (40.4)			13.0 (42.8)	19.9 (42.2)	
SCC	low	31 (66.0)	29 (61.7)	0.830		20.7 (68.1)	32.7 (69.4)	0.925
	high	16 (34.0)	18 (38.3)			9.7 (31.9)	14.4 (30.6)	
Ca199	low	6 (12.8)	13 (27.7)	0.123		4.4 (14.5)	11.3 (24.0)	0.399
	high	41 (87.2)	34 (72.3)			26.0 (85.5)	35.9 (76.0)	
CEA	low	3 (6.4)	13 (27.7)	0.014		2.1 (7.0)	7.0 (14.9)	0.255
	high	44 (93.6)	34 (72.3)			28.3 (93.0)	40.2 (85.1)	
Cyfra211	low	4 (8.5)	14 (29.8)	0.018		4.4 (14.5)	8.3 (17.6)	0.759
	high	43 (91.5)	33 (70.2)			26.0 (85.5)	38.9 (82.4)

**Table:** 

C								
		Unmatched				Stabilized IPTW		
	Level	no-TRT (%)	TRT (%)	*P*		no-TRT (%)	TRT (%)	*P*
Number		15	29			5.1	19.1	
Age	<65	14 (93.3)	21 (72.4)	0.216		4.8 (93.3)	13.8 (72.4)	0.111
	>=65	1 (6.7)	8 (27.6)			0.3 (6.7)	5.3 (27.6)	
Gender	female	8 (53.3)	13 (44.8)	0.828		2.7 (53.3)	8.6 (44.8)	0.599
	male	7 (46.7)	16 (55.2)			2.4 (46.7)	10.5 (55.2)	
KPS	>=80	13 (86.7)	27 (93.1)	0.880		4.4 (86.7)	17.8 (93.1)	0.490
	<80	2 (13.3)	2 (6.9)			0.7 (13.3)	1.3 (6.9)	
Smoking	no	10 (66.7)	19 (65.5)	1.000		3.4 (66.7)	12.5 (65.5)	0.940
	yes	5 (33.3)	10 (34.5)			1.7 (33.3)	6.6 (34.5)	
Histopathology	adenocarcinoma	13 (86.7)	25 (86.2)	1.000		4.4 (86.7)	16.5 (86.2)	0.967
	non-adenocarcinoma	2 (13.3)	4 (13.8)			0.7 (13.3)	2.6 (13.8)	
N stage	N0	2 (13.3)	10 (34.5)	0.256		0.7 (13.3)	6.6 (34.5)	0.144
	N1-3	13 (86.7)	19 (65.5)			4.4 (86.7)	12.5 (65.5)	
T stage	T1-2	11 (73.3)	25 (86.2)	0.524		3.8 (73.3)	16.5 (86.2)	0.304
	T3-4	4 (26.7)	4 (13.8)			1.4 (26.7)	2.6 (13.8)	
Brain metastasis	no	11 (73.3)	25 (86.2)	0.524		3.8 (73.3)	16.5 (86.2)	0.304
	yes	4 (26.7)	4 (13.8)			1.4 (26.7)	2.6 (13.8)	
Bone metastasis	no	4 (26.7)	12 (41.4)	0.528		1.4 (26.7)	7.9 (41.4)	0.346
	yes	11 (73.3)	17 (58.6)			3.8 (73.3)	11.2 (58.6)	
Adrenal metastasis	no	15 (100.0)	28 (96.6)	1.000		5.1 (100.0)	18.5 (96.6)	0.596
	yes	0 (0.0)	1 (3.4)			0.0 (0.0)	0.7 (3.4)	
Liver metastasis	no	14 (93.3)	29 (100.0)	0.734		4.8 (93.3)	19.1 (100.0)	0.059
	yes	1 (6.7)	0 (0.0)			0.3 (6.7)	0.0 (0.0)	
SM status	SM+	14 (93.3)	18 (62.1)	0.064		4.8 (93.3)	11.9 (62.1)	0.031
	SM-	1 (6.7)	11 (37.9)			0.3 (6.7)	7.3 (37.9)	
LT to metastatic sites before PD	all	5 (33.3)	17 (58.6)	0.203		1.7 (33.3)	11.2 (58.6)	0.120
	partly or no	10 (66.7)	12 (41.4)			3.4 (66.7)	7.9 (41.4)	
Albumin	high	0 (0.0)	5 (17.2)	0.227		0.0 (0.0)	3.3 (17.2)	0.182
	low	15 (100.0)	24 (82.8)			5.1 (100.0)	15.8 (82.8)	
Leukocyte	low	12 (80.0)	20 (69.0)	0.673		4.1 (80.0)	13.2 (69.0)	0.445
	high	3 (20.0)	9 (31.0)			1.0 (20.0)	5.9 (31.0)	
PLR	low	5 (33.3)	9 (31.0)	1.000		1.7 (33.3)	5.9 (31.0)	0.879
	high	10 (66.7)	20 (69.0)			3.4 (66.7)	13.2 (69.0)	
NLR	low	3 (20.0)	6 (20.7)	1.000		1.0 (20.0)	4.0 (20.7)	0.958
	high	12 (80.0)	23 (79.3)			4.1 (80.0)	15.2 (79.3)	
SII	low	3 (20.0)	5 (17.2)	1.000		1.0 (20.0)	3.3 (17.2)	0.825
	high	12 (80.0)	24 (82.8)			4.1 (80.0)	15.8 (82.8)	
TPSA	low	10 (66.7)	26 (89.7)	0.144		3.4 (66.7)	17.1 (89.7)	0.069
	high	5 (33.3)	3 (10.3)			1.7 (33.3)	2.0 (10.3)	
SCC	low	14 (93.3)	24 (82.8)	0.613		4.8 (93.3)	15.8 (82.8)	0.342
	high	1 (6.7)	5 (17.2)			0.3 (6.7)	3.3 (17.2)	
Ca199	low	4 (26.7)	10 (34.5)	0.852		1.4 (26.7)	6.6 (34.5)	0.604
	high	11 (73.3)	19 (65.5)			3.8 (73.3)	12.5 (65.5)	
CEA	low	2 (13.3)	4 (13.8)	1.000		0.7 (13.3)	2.6 (13.8)	0.967
	high	13 (86.7)	25 (86.2)			4.4 (86.7)	16.5 (86.2)	
Cyfra211	low	4 (26.7)	20 (69.0)	0.019		1.4 (26.7)	13.2 (69.0)	0.011
	high	11 (73.3)	9 (31.0)			3.8 (73.3)	5.9 (31.0)

**Table:** 

D								
		Unmatched				Stabilized IPTW		
	Level	no-TRT (%)	TRT (%)	*P*		no-TRT (%)	TRT (%)	*P*
Number		31	20			27.8	16.2	
Age	<65	18 (58.1)	12 (60.0)	1.000		14.1 (50.8)	7.1 (43.6)	0.665
	>=65	13 (41.9)	8 (40.0)			13.7 (49.2)	9.1 (56.4)	
Gender	female	8 (25.8)	8 (40.0)	0.449		7.4 (26.5)	5.3 (32.4)	0.678
	male	23 (74.2)	12 (60.0)			20.4 (73.5)	11.0 (67.6)	
KPS	>=80	29 (93.5)	18 (90.0)	1.000		26.3 (94.7)	15.3 (94.2)	0.937
	<80	2 (6.5)	2 (10.0)			1.5 (5.3)	0.9 (5.8)	
Smoking	no	11 (35.5)	10 (50.0)	0.461		10.0 (36.2)	6.3 (38.7)	0.871
	yes	20 (64.5)	10 (50.0)			17.7 (63.8)	9.9 (61.3)	
Histopathology	adenocarcinoma	23 (74.2)	10 (50.0)	0.143		19.0 (68.5)	9.9 (60.9)	0.640
	non-adenocarcinoma	8 (25.8)	10 (50.0)			8.8 (31.5)	6.3 (39.1)	
N stage	N0	2 (6.5)	4 (20.0)	0.307		4.1 (14.6)	3.2 (19.5)	0.739
	N1-3	29 (93.5)	16 (80.0)			23.7 (85.4)	13.0 (80.5)	
T stage	T1-2	15 (48.4)	6 (30.0)	0.312		13.6 (48.8)	6.4 (39.6)	0.598
	T3-4	16 (51.6)	14 (70.0)			14.2 (51.2)	9.8 (60.4)	
Brain metastasis	no	28 (90.3)	18 (90.0)	1.000		25.6 (92.3)	15.2 (94.0)	0.788
	yes	3 (9.7)	2 (10.0)			2.1 (7.7)	1.0 (6.0)	
Bone metastasis	no	17 (54.8)	14 (70.0)	0.430		14.1 (50.7)	9.6 (59.2)	0.622
	yes	14 (45.2)	6 (30.0)			13.7 (49.3)	6.6 (40.8)	
Adrenal metastasis	no	25 (80.6)	18 (90.0)	0.615		23.1 (83.0)	13.2 (81.7)	0.928
	yes	6 (19.4)	2 (10.0)			4.7 (17.0)	3.0 (18.3)	
SM status	SM+	5 (16.1)	2 (10.0)	0.838		5.1 (18.2)	3.4 (21.1)	0.854
	SM-	26 (83.9)	18 (90.0)			22.7 (81.8)	12.8 (78.9)	
LT to metastatic sites before PD	all	2 (6.5)	8 (40.0)	0.010		1.9 (6.8)	3.5 (21.5)	0.124
	partly or no	29 (93.5)	12 (60.0)			25.9 (93.2)	12.7 (78.5)	
Albumin	high	5 (16.1)	3 (15.0)	1.000		5.0 (18.1)	3.7 (23.0)	0.750
	low	26 (83.9)	17 (85.0)			22.7 (81.9)	12.5 (77.0)	
Leukocyte	low	17 (54.8)	9 (45.0)	0.690		16.3 (58.6)	8.2 (50.9)	0.642
	high	14 (45.2)	11 (55.0)			11.5 (41.4)	8.0 (49.1)	
PLR	low	4 (12.9)	2 (10.0)	1.000		5.3 (19.0)	3.5 (21.6)	0.873
	high	27 (87.1)	18 (90.0)			22.5 (81.0)	12.7 (78.4)	
NLR	low	2 (6.5)	1 (5.0)	1.000		3.3 (12.1)	1.8 (11.2)	0.950
	high	29 (93.5)	19 (95.0)			24.4 (87.9)	14.4 (88.8)	
SII	low	2 (6.5)	1 (5.0)	1.000		3.3 (12.1)	1.8 (11.2)	0.950
	high	29 (93.5)	19 (95.0)			24.4 (87.9)	14.4 (88.8)	
TPSA	low	14 (45.2)	12 (60.0)	0.454		12.3 (44.2)	7.8 (48.4)	0.806
	high	17 (54.8)	8 (40.0)			15.5 (55.8)	8.4 (51.6)	
SCC	low	21 (67.7)	14 (70.0)	1.000		20.4 (73.3)	11.5 (71.1)	0.888
	high	10 (32.3)	6 (30.0)			7.4 (26.7)	4.7 (28.9)	
Ca199	low	7 (22.6)	8 (40.0)	0.309		7.5 (27.2)	4.7 (28.8)	0.912
	high	24 (77.4)	12 (60.0)			20.2 (72.8)	11.5 (71.2)	
CEA	low	4 (12.9)	5 (25.0)	0.465		4.8 (17.1)	2.5 (15.6)	0.899
	high	27 (87.1)	15 (75.0)			23.0 (82.9)	13.7 (84.4)	
Cyfra211	low	6 (19.4)	4 (20.0)	1.000		4.9 (17.8)	2.4 (15.1)	0.802
	high	25 (80.6)	16 (80.0)			22.8 (82.2)	13.8 (84.9)	

KPS, Karnofsky performance status; SM, sensitive mutation; LT, local treatment; PD, progress disease; TRT, thoracic radiotherapy; PLR, platelet to lymphocyte ratio; NLR, neutrophils to lymphocyte ratio; SII, systemic inflammatory index; TPSA, tissue polypeptide specific antigen; SCC, squamous cell carcinoma antigen; CEA, carcinoembryonic antigen.

**Figure 3:**
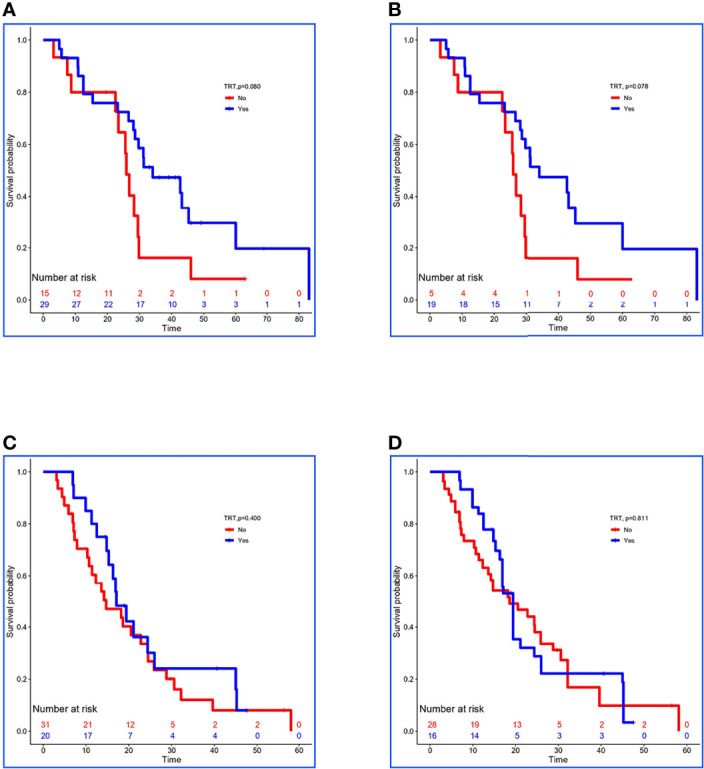
Kaplan–Meier survival analyses for patients between groups. **(A, B)** Survival curves for low-risk patients between non-TRT and TRT groups when clinical characteristics were unmatched and matched using stabilized IPTW analysis in the validation set. **(C, D)** Survival curves for high-risk patients between non-TRT and TRT groups when clinical characteristics were unmatched and matched using stabilized IPTW analysis in the validation set.

In the validation set, 29 of 44 low-risk patients received TRT, and the OS was prolonged 8.2 months (34.1 vs. 25.9 months, *P =* 0.080; **Figure 3A**). In addition, stabilized IPTW analysis was used to match the clinical characteristics (**Table 6C**), and the between-group differences in OS were close to statistical as ever (34.1 vs. 25.9 months, *P =* 0.078; **Figure 3B**). Meanwhile, 51 patients were divided into high-risk group, and 20 of them received TRT with no improvement in OS (17.1 vs. 14.7 months, *P =* 0.400; **Figure 3C**). On the basis of the clinical characteristics, the TRT group had more patients with no treatment to metastatic sites, which may have influenced the result (**Table 6D**). Similarly, we applied stabilized IPTW analysis to match the groups. After matching, TRT was not found to have improved survival as before (19.4 vs. 18.6 months, *P =* 0.811; **Figure 3D**).

## Discussion

In the current study, we established a risk prediction model to predict the mortality risk of patients with SOM-NSCLC and, further, to identify patients who would not benefit from TRT. Eventually, a total of 283 cases met the inclusion criteria and were divided into the training and validation sets. A Risk-Total formula constructed by 10 clinical prognostic factors was used to calculate each patient’s risk score, and patients were divided into low- and high-risk groups according to the median value (10.0658) in the training set. Then, TRT was found to just have improved the survival of low-risk patients (*P =* 0.083) but not that of high-risk patients (*P =* 0.663) in the training set. Similarly, patients in the validation set were estimated risk-score on the basis of the Risk-Total formula, and were grouped into low- and high-risk groups basing on the median value (10.0658), and TRT only prolonged the OS of low-risk patients (*P =* 0.078) but not that of the high-risk patients (*P =* 0.811).

The biological characteristics of oligometastatic cancer are increasingly being defined, and the role of LT has evolved substantially during the past decade. In 2018, a prospective, multicenter, single-arm, phase 2 trial reported the long-term outcomes of consolidative radiation therapy (CRT) to the primary and metastatic sites from oligometastatic NSCLC, achieving a partial response or stable disease after three to six cycles of platinum-based chemotherapy. The median PFS and OS were 11.2 and 28.4 months, respectively, which met the primary endpoint and transcended the historical record (13). The first multicenter randomized trial of local consolidative therapy (LCT) for highly selected oligometastatic NSCLC (≤3 metastatic lesions, no progression after front-line systemic therapy) demonstrated significant PFS (14.2 vs. 4.4 months) and OS (41.2 vs. 17.0 months) benefit compared with patients who received maintenance therapy or observation (8). Another single-center randomized phase 2 study of maintenance chemotherapy alone versus stereotactic ablative radiotherapy followed by maintenance chemotherapy for patients with limited metastatic NSCLC (primary plus up to five metastatic sites) with no *EGFR*-targetable or *ALK*-targetable mutations but who did achieve a partial response or stable disease after induction chemotherapy also obtained gratifying results (7). Despite differences in the population inclusion criteria in these clinical trials, there was significant prolongation of OS (range of 28.4–41.2 months). However, some patients with SOM-NSCLC experienced rapid disease progression after TRT and showed no significant survival benefit. However, to date, no effective predictive model has been developed to help identify patients who would not benefit from TRT. Hence, in the present study, we established a risk prediction model to predict the mortality risk of patients with SOM-NSCLC and, further, to identify patients who would not benefit from TRT.

Several hematological and clinical factors have been shown to suggest a bad prognosis for lung cancer including hypoalbuminemia (16–18); increase of C-reactive protein (18, 19), lactate dehydrogenase (20), PLR (17, 21–23), NLR (17, 21–24), SII (17, 21), and tumor biomarkers (20, 25); abnormal coagulation and fibrinolysis (26, 27); high T and N stage; liver metastasis; adrenal metastasis (28, 29); absence of SMs; smoking history; male; and loss of weight (30). In the present study, 10 variables were included in the Risk-Total formula, and the level of risk score was associated with reduced survival of patients, which was consistent with previous studies. According to this model, we found that TRT just improve the survival of low-risk patients but not that of high-risk.

In recent years, immunotherapy has transformed the treatment approach for patients with advanced NSCLC. The combination of immunotherapy and LCT for these potentially curable patients is an area of active investigation. Bauml et al. (31) randomized 51 patients who had oligometastatic NSCLC (≤4 metastatic sites) and had completed LT to all known sites of disease to receive pembrolizumab. The median PFS was significantly greater than historical data (*P =* 0.005), and 1- and 2-year OS rates were 90.9% and 77.5%, respectively. Nevertheless, in our study, immunotherapy status was not included in the analysis, which may affect the practicality of this prediction model in the era of immunotherapy.

## Limitations

Some limitations of our study should be considered. Most importantly, because of the retrospective study design, the diagnosis of metastatic sites was not based on homogenous imaging techniques. Next, local and systematic treatments were also inconsistent, which may have influenced survival. Finally, this study was based on the experience of a single institution, and the number of patients was limited. Future multicenter studies are required to verify this model and to refine the treatment method for primary lesion.

## Conclusion

The prognosis of SOM-NSCLC is significantly influenced by many hematological and clinical factors. A prediction model was developed in this study to help identify patients who would not benefit from TRT, and we found that TRT improved the survival of low-risk patients but not that of the high-risk patients.

## Data Availability Statement

The original contributions presented in the study are included in the article/supplementary material. Further inquiries can be directed to the corresponding authors.

## Ethics Statement

The studies involving human participants were reviewed and approved by Department of Ethics Committee, Tianjin Medical University Cancer Institute and Hospital. Written informed consent for participation was not required for this study in accordance with the national legislation and the institutional requirements.

## Author Contributions

CM: Conceptualization, Methodology, Formal analysis, Investigation, Writing - Original Draft. FW: Conceptualization, Methodology, Formal analysis, Investigation. JT, JW, and XL: Investigation. KR and LX: Methodology. LZ and PW: Writing - Review and Editing. All authors contributed to the article and approved the submitted version.

## Funding

This work was supported by the Chinese National Key Research and Development Project (Grant No. 2018YFC1315601), and the National Natural Science Foundation of China (No.81903121).

## Conflict of Interest

The authors declare that the research was conducted in the absence of any commercial or financial relationships that could be construed as a potential conflict of interest.

## Publisher’s Note

All claims expressed in this article are solely those of the authors and do not necessarily represent those of their affiliated organizations, or those of the publisher, the editors and the reviewers. Any product that may be evaluated in this article, or claim that may be made by its manufacturer, is not guaranteed or endorsed by the publisher.

## Footnotes

2
^2^***Abbreviations***: NSCLC, non–small cell lung cancer; LT, local treatment; TRT, thoracic radiotherapy; OS, overall survival; PFS, progression-free survival; TNM, tumor node metastasis; SM, sensitive mutations; EGFR, epidermal growth factor receptor; ALK, anaplastic lymphoma kinase; ROS1, ROS proto-oncogene 1, receptor tyrosine kinase; TPSA, tissue polypeptide–specific antigen; SCC, squamous cell carcinoma antigen; CEA, carcinoembryonic antigen; PLR, platelet-to-lymphocyte ratio; NLR, neutrophil-to-lymphocyte ratio; SII, systemic inflammatory index; LASSO, least absolute shrinkage and selection operator; MST, median survival time; ROC, receiver operator characteristic; AUC, area under the curve; KPS, Karnofsky performance status; SBRT, stereotactic body radiotherapy; IPTW, inverse probability treatment weight.

## Abbreviations

NSCLC, non–small cell lung cancer; LT, local treatment; TRT, thoracic radiotherapy; OS, overall survival; PFS, progression-free survival; TNM, tumor node metastasis; SM, sensitive mutations; EGFR, epidermal growth factor receptor; ALK, anaplastic lymphoma kinase; ROS1, ROS proto-oncogene 1, receptor tyrosine kinase; TPSA, tissue polypeptide–specific antigen; SCC, squamous cell carcinoma antigen; CEA, carcinoembryonic antigen; PLR, platelet-to-lymphocyte ratio; NLR, neutrophil-to-lymphocyte ratio; SII, systemic inflammatory index; LASSO, least absolute shrinkage and selection operator; MST, median survival time; ROC, receiver operator characteristic; AUC, area under the curve; KPS, Karnofsky performance status; SBRT, stereotactic body radiotherapy; IPTW, inverse probability treatment weight.

## References

[1] Sung H Ferlay J Siegel RL Laversanne M Soerjomataram I Jemal A. Global Cancer Statistics 2020: GLOBOCAN Estimates of Incidence and Mortality Worldwide for 36 Cancers in 185 Countries. CA Cancer J Clin (2021) 71(3):209–249. doi: 10.3322/caac.21660 33538338

[2] Levy A Hendriks LEL Berghmans T Faivre-Finn C GiajLevra M GiajLevra N. EORTC Lung Cancer Group Survey on the Definition of NSCLC Synchronous Oligometastatic Disease. Eur J Cancer (2019) 122:109–14. doi: 10.1016/j.ejca.2019.09.012 31671363

[3] Parikh RB Cronin AM Kozono DE Oxnard GR Mak RH Jackman DM. Definitive Primary Therapy in Patients Presenting With Oligometastatic non-Small Cell Lung Cancer. Int J Radiat Oncol Biol Phys (2014) 89(4):880–7. doi: 10.1016/j.ijrobp.2014.04.007 24867533

[4] Hellman S Weichselbaum RR. Oligometastases. J Clin Oncol (1995) 13(1):8–10. doi: 10.1200/JCO.1995.13.1.8 7799047

[5] Lievens Y Guckenberger M Gomez D Hoyer M Iyengar P Kindts I. Defining Oligometastatic Disease From a Radiation Oncology Perspective: An ESTRO-ASTRO Consensus Document. Radiother Oncol (2020) 148:157–66. doi: 10.1016/j.radonc.2020.04.003 32388150

[6] Xu Q Zhou F Liu H Jiang T Li X Xu Y. Consolidative Local Ablative Therapy Improves the Survival of Patients With Synchronous Oligometastatic NSCLC Harboring EGFR Activating Mutation Treated With First-Line EGFR-TKIs. J Thorac Oncol (2018) 13(9):1383–92. doi: 10.1016/j.jtho.2018.05.019 29852232

[7] Iyengar P Wardak Z Gerber DE Tumati V Ahn C Hughes RS. Consolidative Radiotherapy for Limited Metastatic Non-Small-Cell Lung Cancer: A Phase 2 Randomized Clinical Trial. JAMA Oncol (2018) 4(1):e173501. doi: 10.1001/jamaoncol.2017.3501 28973074 PMC5833648

[8] Gomez D Tang C Zhang J Blumenschein G Hernandez M Lee J. Local Consolidative Therapy Vs. Maintenance Therapy or Observation for Patients With Oligometastatic Non-Small-Cell Lung Cancer Long-Term Results of a Multi-Institutional, Phase II, Randomized Study. J Clin Oncol (2019) 37(18):1558–65. doi: 10.1200/JCO.19.00201 PMC6599408 31067138

[9] Palma DA Olson R Harrow S Gaede S Louie AV Haasbeek C. Stereotactic Ablative Radiotherapy Versus Standard of Care Palliative Treatment in Patients With Oligometastatic Cancers (SABR-COMET): A Randomised, Phase 2, Open-Label Trial. Lancet (2019) 393(10185):2051–8. doi: 10.1016/S0140-6736(18)32487-5 30982687

[10] Lopez Guerra JL Gomez D Zhuang Y Hong DS Heymach JV Swisher SG. Prognostic Impact of Radiation Therapy to the Primary Tumor in Patients With non-Small Cell Lung Cancer and Oligometastasis at Diagnosis. Int J Radiat Oncol Biol Phys (2012) 84(1):e61–7. doi: 10.1016/j.ijrobp.2012.02.054 PMC3919541 22503522

[11] Ashworth AB Senan S Palma DA Riquet M Ahn YC Ricardi U. An Individual Patient Data Metaanalysis of Outcomes and Prognostic Factors After Treatment of Oligometastatic non-Small-Cell Lung Cancer. Clin Lung Cancer (2014) 15(5):346–55. doi: 10.1016/j.cllc.2014.04.003 24894943

[12] Collen C Christian N Schallier D Meysman M Duchateau M Storme G. Phase II Study of Stereotactic Body Radiotherapy to Primary Tumor and Metastatic Locations in Oligometastatic Nonsmall-Cell Lung Cancer Patients. Ann Oncol (2014) 25(10):1954–9. doi: 10.1093/annonc/mdu370 25114022

[13] Petty WJ Urbanic JJ Ahmed T Hughes R Levine B Rusthoven K. Long-Term Outcomes of a Phase 2 Trial of Chemotherapy With Consolidative Radiation Therapy for Oligometastatic Non-Small Cell Lung Cancer. Int J Radiat Oncol Biol Phys (2018) 102(3):527–35. doi: 10.1016/j.ijrobp.2018.06.400 PMC6743975 30003996

[14] Farooqi A Ludmir EB Mitchell KG Antonoff MB Tang C Lee P. Increased Biologically Effective Dose (BED) to the Primary Tumor is Associated With Improved Survival in Patients With Oligometastatic NSCLC. Radiother Oncol (2021) 163:114–8. doi: 10.1016/j.radonc.2021.08.005 34419505

[15] Chansky K Detterbeck FC Nicholson AG Rusch VW Vallieres E Groome P. The IASLC Lung Cancer Staging Project: External Validation of the Revision of the TNM Stage Groupings in the Eighth Edition of the TNM Classification of Lung Cancer. J Thorac Oncol (2017) 12(7):1109–21. doi: 10.1016/j.jtho.2017.04.011 28461257

[16] Meng C Wei J Tian J Ma J Liu N Yuan Z. Estimating Survival and Clinical Outcome in Advanced non-Small Cell Lung Cancer With Bone-Only Metastasis Using Molecular Markers. J Bone Oncol (2021) 31:100394. doi: 10.1016/j.jbo.2021.100394 34703756 PMC8524192

[17] Qi J Zhang J Ge X Wang X Xu L Liu N. The Addition of Peripheral Blood Inflammatory Indexes to Nomogram Improves the Predictive Accuracy of Survival in Limited-Stage Small Cell Lung Cancer Patients. Front Oncol (2021) 11:713014. doi: 10.3389/fonc.2021.713014 34692490 PMC8531548

[18] Ni XF Wu J Ji M Shao YJ Xu B Jiang JT. Effect of C-Reactive Protein/Albumin Ratio on Prognosis in Advanced non-Small-Cell Lung Cancer. Asia Pac J Clin Oncol (2018) 14(6):402–9. doi: 10.1111/ajco.13055 30178541

[19] Cetin K Christiansen CF Jacobsen JB Norgaard M Sorensen HT. Bone Metastasis, Skeletal-Related Events, and Mortality in Lung Cancer Patients: A Danish Population-Based Cohort Study. Lung Cancer (2014) 86(2):247–54. doi: 10.1016/j.lungcan.2014.08.022 25240518

[20] Gu W Hu M Xu L Ren Y Mei J Wang W. The Ki-67 Proliferation Index-Related Nomogram to Predict the Response of First-Line Tyrosine Kinase Inhibitors or Chemotherapy in Non-Small Cell Lung Cancer Patients With Epidermal Growth Factor Receptor-Mutant Status. Front Med (Lausanne) (2021) 8:728575. doi: 10.3389/fmed.2021.728575 34805200 PMC8602562

[21] Tong YS Tan J Zhou XL Song YQ Song YJ. Systemic Immune-Inflammation Index Predicting Chemoradiation Resistance and Poor Outcome in Patients With Stage III non-Small Cell Lung Cancer. J Transl Med (2017) 15(1):221. doi: 10.1186/s12967-017-1326-1 29089030 PMC5664920

[22] Chen C Yang H Cai D Xiang L Fang W Wang R. Preoperative Peripheral Blood Neutrophil-to-Lymphocyte Ratios (NLR) and Platelet-to-Lymphocyte Ratio (PLR) Related Nomograms Predict the Survival of Patients With Limited-Stage Small-Cell Lung Cancer. Trans Lung Cancer Res (2021) 10(2):866–77. doi: 10.21037/tlcr-20-997 PMC7947425 33718028

[23] Zhang N Jiang J Tang S Sun G. Predictive Value of Neutrophil-Lymphocyte Ratio and Platelet-Lymphocyte Ratio in Non-Small Cell Lung Cancer Patients Treated With Immune Checkpoint Inhibitors: A Meta-Analysis. Int Immunopharmacol (2020) 85:106677. doi: 10.1016/j.intimp.2020.106677 32531712

[24] Huang W Wang S Zhang H Zhang B Wang C. Prognostic Significance of Combined Fibrinogen Concentration and Neutrophil-to-Lymphocyte Ratio in Patients With Resectable non-Small Cell Lung Cancer. Cancer Biol Med (2018) 15(1):88–96. doi: 10.20892/j.issn.2095-3941.2017.0124 29545972 PMC5842339

[25] Dall'Olio FG Abbati F Facchinetti F Massucci M Melotti B Squadrilli A. CEA and CYFRA 21-1 as Prognostic Biomarker and as a Tool for Treatment Monitoring in Advanced NSCLC Treated With Immune Checkpoint Inhibitors. Ther Adv Med Oncol (2020) 12:1758835920952994. doi: 10.1177/1758835920952994 33193825 PMC7607728

[26] Bharadwaj AG Holloway RW Miller VA Waisman DM. Plasmin and Plasminogen System in the Tumor Microenvironment: Implications for Cancer Diagnosis, Prognosis, and Therapy, Cancers (Basel) 13(8):1838. (2021). doi: 10.3390/cancers13081838 PMC8070608 33921488

[27] Guo J Gao Y Gong Z Dong P Mao Y Li F. Plasma D-Dimer Level Correlates With Age, Metastasis, Recurrence, Tumor-Node-Metastasis Classification (TNM), and Treatment of Non-Small-Cell Lung Cancer (NSCLC) Patients. BioMed Res Int (2021) 2021:9623571. doi: 10.1155/2021/9623571 34712737 PMC8548094

[28] Nakazawa K Kurishima K Tamura T Kagohashi K Ishikawa H Satoh H. Specific Organ Metastases and Survival in Small Cell Lung Cancer. Oncol Lett (2012) 4(4):617–20. doi: 10.3892/ol.2012.792 PMC3506697 23205072

[29] Riihimäki M Hemminki A Fallah M Thomsen H Sundquist K Sundquist J. Metastatic Sites and Survival in Lung Cancer. Lung Cancer (2014) 86(1):78–84. doi: 10.1016/j.lungcan.2014.07.020 25130083

[30] Wang J Zhao Y Wang Q Zhang L Shi J Wang Z. Prognostic Factors of Refractory NSCLC Patients Receiving Anlotinib Hydrochloride as the Third- or Further-Line Treatment. Cancer Biol Med (2018) 15(4):443–51. doi: 10.20892/j.issn.2095-3941.2018.0158 PMC6372914 30766754

[31] Bauml JM Mick R Ciunci C Aggarwal C Davis C Evans T. Pembrolizumab After Completion of Locally Ablative Therapy for Oligometastatic Non-Small Cell Lung Cancer: A Phase 2 Trial. JAMA Oncol (2019) 5(9):1283–90. doi: 10.1001/jamaoncol.2019.1449 PMC6624820 31294762

